# A novel PDHK inhibitor restored cognitive dysfunction and limited neurodegeneration without affecting amyloid pathology in 5xFAD mouse, a model of Alzheimer’s disease

**DOI:** 10.1186/s13195-024-01552-2

**Published:** 2024-09-05

**Authors:** Katsuya Sakimura, Takashi Kawai, Reiko Nashida, Yuji Ishida, Kana Harada, Takashi Suzuki, Chihiro Okuma, Gregory M. Cole

**Affiliations:** 1 Biological/Pharmacological Research Laboratories, Central Pharmaceutical Research Institute, Japan Tobacco Inc, 1-1 Murasaki-cho, Takatsuki, Osaka Japan; 2 Department of Neurology & Medicine, Veterans Affairs Healthcare System, GRECC, David Geffen School of Medicine at UCLA, Los Angeles, USA

**Keywords:** Alzheimer’s disease, Pyruvate dehydrogenase (PDH), Pyruvate dehydrogenase kinase (PDHK), 5xFAD, Glucose metabolism, Aerobic glycolysis

## Abstract

**Background:**

Alzheimer’s disease (AD) is the most common form of dementia. Although drugs focusing on reducing amyloid β slow progression, they fail to improve cognitive function. Deficits in glucose metabolism are reflected in FDG-PET and parallel the neurodegeneration and synaptic marker loss closely preceding cognitive decline, but the role of metabolic deficits as a cause or consequence of neurodegeneration is unclear. Pyruvate dehydrogenase (PDH) is lost in AD and an important enzyme connecting glycolysis and the tricarboxylic acid (TCA) cycle by converting pyruvate into acetyl-CoA. It is negatively regulated by pyruvate dehydrogenase kinase (PDHK) through phosphorylation.

**Methods:**

In the present study, we assessed the in vitro/ in vivo pharmacological profile of the novel PDHK inhibitor that we discovered, Compound A. We also assessed the effects of Compound A on AD-related phenotypes including neuron loss and cognitive impairment using 5xFAD model mice.

**Results:**

Compound A inhibited human PDHK1, 2 and 3 but had no inhibitory activity on PDHK4. In primary neurons, Compound A enhanced pyruvate and lactate utilization, but did not change glucose levels. In contrast, in primary astrocytes, Compound A enhanced pyruvate and glucose utilization and enhanced lactate production. In an efficacy study using 5xFAD mice, Compound A ameliorated the cognitive dysfunction in the novel object recognition test and Morris water maze. Moreover, Compound A prevented neuron loss in the hippocampus and cerebral cortex of 5xFAD without affecting amyloid β deposits.

**Conclusions:**

These results suggest ameliorating metabolic deficits by activating PDH by Compound A can limit neurodegeneration and is a promising therapeutic strategy for treating AD.

**Supplementary Information:**

The online version contains supplementary material available at 10.1186/s13195-024-01552-2.

## Introduction

Alzheimer’s disease (AD) is the most common form of dementia and accounts for 60–80% of cases. AD is a progressive neurodegenerative disease, irreversible and disabling, causing a large socioeconomic burden. The most common symptom present at the beginning of AD is associated with short term memory deficit, which affects daily activities. Pathologically, AD involves progressive deposition of insoluble amyloid β (Aβ) peptide as amyloid plaques, intracellular accumulation of hyperphosphorylated tau protein as neurofibrillary tangles and neuron and synapse loss. Until recently, there were only two classes of drugs approved for the treatment of AD, cholinesterase inhibitors and N-methyl-D aspartate (NMDA) receptor antagonists. These drugs improve symptoms with limited efficacy and do not cure or prevent the disease progression [1–3]. Recently, Aduhelm, Lecanemab and Donanemab anti-Aβ antibodies have been shown to effectively reduce patient amyloid burden and modify disease pathology. They can significantly slow the disease progression; however, it should be noted that their efficacy was only demonstrated for patients with mild cognitive impairment or mild AD [4]. Thus, beyond amyloid reduction, drugs with additional mechanism of actions, for example targeting tauopathy or metabolic deficits are still needed to effectively treat or prevent AD.

A growing body of evidence argues that cerebral glucose hypometabolism is a hallmark of AD which is closely correlated with progressive disease severity. Consistent with this, energy production in AD brain is shifted toward metabolizing alternative fuels, including amino acids, lipids and ketone bodies. Altered expression and/or activities of many glucose metabolism-related enzymes have been reported in postmortem AD brains. For example, α-ketoglutarate dehydrogenase (KGDH) activity was reduced while that of succinate dehydrogenase (SDH) and malate dehydrogenase (MDH) were increased in AD. The activity of pyruvate dehydrogenase (PDH), the rate-limiting enzyme for pyruvate entry into tricarboxylic acid (TCA) cycle was also decreased, and further, the degree of decrease has been correlated with the CDR, a measure of cognitive function in AD [5]. Therefore, we hypothesized that activating PDH might be a promising therapeutic strategy for treating the deficits in glucose metabolism in AD.

PDH is a multi-enzyme complex consisting of three enzymatic components (E1, E2 and E3) localized in the mitochondrial matrix. PDH provides substrates for aerobic glucose oxidation (TCA cycle and electron transfer system) by converting pyruvate to acetyl-Coenzyme A (acetyl-CoA). Thus, PDH plays an important role in regulating the coupling between glycolysis and glucose oxidation and control of both energy (Adenosine tri-phosphate (ATP)) and acetyl-CoA, an important regulatory molecule. Patients with PDH deficiency are known to have abnormal brain structure and many neurological impairments including cognitive dysfunction. Consistent with this, brain-specific PDH-deficient mice exhibit neuron loss in several brain regions and develop functional deficits [6], consistent with similar deficits with more generalized deficiency [7, 8]. Collectively, the literature argues that PDH plays a major role in the central nervous system that affects functional status. Pyruvate dehydrogenase kinase (PDHK), a serine/threonine kinase specific for PDH, is known to regulate PDH negatively via phosphorylation of the E1α subunit of PDH complex. Four isoforms of PDHK have been identified to date, all of which are expressed in neurons and glial cells in the human brain [9]. Therefore, inhibition of PDHK can increase PDH activity followed by enhancing utilization of glucose and pyruvate.

Compound A is a novel potent PDHK inhibitor discovered by screening based on its inhibitory activity against human PDHK1, 2 and 3. In the present study, we investigated the effects of PDHK inhibition on brain glucose metabolism using Compound A. We also examined treatment effects in 5xFAD model mice to assess the therapeutic potential of PDHK inhibition as an AD treatment.

## Materials and methods

### Animals

Except for the 5xFAD studies, all experiments were performed using Sprague-Dawley (SD) rats at 8 to 12 weeks of age (Charles River Japan, Tokyo, Japan). The rats were group housed with 2–3 per cage in a climate-controlled room with a temperature of 23 °C ± 3 °C, and a 12-h dark-light cycle, with food and water ad libitum. These experiments were conducted in compliance with the Guidelines for Animal Experimentation of Japan Tobacco Biological/ Pharmacological Research Laboratories.

For AD model mice studies, 5xFAD mice (APPSwFlLon, PSEN1*M146L*L286V) 6799Vas/Mmjax) and control B6SJL mice were purchased from Jackson Laboratories and maintained by the UCLA Division of Laboratory Animal Medicine. Mice were housed in groups of 5, under a 12-h light/12-h dark cycle at 21 °C, with food and water ad libitum. All the experiments were reviewed and carried out in accordance with National Research Council Guide for the Care and Use of Laboratory Animals and approved by the University of California, Los Angeles Institutional Animal Care Use Committee, and performed with strict adherence to the guidelines set out in the National Institutes of Health Guide for the Care and Use of Laboratory Animals.

### Inhibitory activity of compound A

For assessing the inhibitory activity of Compound A on human PDHK (hPDHK) and rat PDHK (rPDHK), recombinant hPDHK1-4, rPDHK1-4 with a Flag-tag at the N-terminus and PDH enzyme derived from porcine heart were used. PDHK activity was assessed indirectly by measuring the residual PDH activity after PDHK reaction as described previously [10]. Briefly, PDH (P7032, Sigma-Aldrich, STL, USA) and human/ rat PDHK1, 2, 3 or 4 (Central Pharmaceutical Research Institute, Japan Tobacco Inc.) were mixed and incubated in a refrigerator at 4 °C overnight to obtain a PDH/PDHK complex. The PDH/PDHK complex and Compound A were incubated for 45 min at room temperature in a 96-well half area microplate after adding 0.3 µM ATP (for PDHK1, 2 and 3) or 10 µM ATP (for PDHK4) to start the PDHK reaction. Then, the substrates for PDH in reaction buffer (50 mM MOPS (pH 7.0), 20 mM K_2_HPO_4_, 60 mM KCl, 2 mM MgCl_2_, 0.4 mM EDTA, 0.2% Pluronic F-68, 2 mM DTT, 5 mM CoA, 5 mM Sodium pyruvate, 5 mM TPP and 12 mM β-nicotinamide adenine dinucleotide) were added to start the PDH reaction and incubated for 90 min at room temperature. The absorbance at 340 nm before and after the PDH reaction was measured to determine the production of NADH by the PDH reaction. Three independent trials were performed and the IC_50_ value of Compound A was calculated for each type of PDHK enzyme. The PDHK inhibition (%) of the test compound, a reversible inhibitor, was calculated from the formula [{(PDH activity of test compound − PDH activity of control)/(PDH activity of blank − PDH activity of control)} × 100]. The IC_50_ value was calculated from the concentration of the test compound at the two points between the PDHK inhibition rates of 50%.

### Rat primary cultures and drug treatment

Primary cortical neurons were prepared from embryonic day 16 SD (IGS) rat embryos (Charles River Laboratories Japan). Briefly, cortical fragments of 8 litters were dissociated into single cells with a Neuron Dissociation Solutions kit (Cat No. 291-78001, FUJIFILM Wako Pure Chemical Corporation, Japan) and resuspended in Neurobasal medium (Cat No. 21103-049, Thermo Fisher Scientific, USA) supplemented with 2% B27 (Cat No. 17504-044, Thermo Fisher Scientific, USA). The neurons were seeded onto poly-L-lysine-coated 96-well plates (Cat No. 356516, Corning, NY 14831 USA) at a density of 80,000 cells per well and cultured in Neurobasal medium supplemented with 2% B27 for 10 days to perform the assay in a 5% CO2/95% air atmosphere. On Day 10, half of the culture medium was removed and collected into a new 96-well plate. The new medium containing the DMSO or Compound A at double the final concentration was added onto both plates, and the plates without cells were designated as pre-treatment media samples. After the cells were incubated for 24 h, the medium was collected as the post-treatment samples.

Primary astrocyte cultures were prepared from the cortex of 3 pups of SD (IGS) rats at postnatal day 1. Briefly, the cells were isolated with a Neuron Dissociation Solutions kit, as described above, and then cultured in DMEM (Cat No. 10569-010, Thermo Fisher Scientific, USA) with 10% heat-inactivated FBS (Cat No. 16000-077,Thermo Fisher Scientific, USA) and 1% N-2 (Cat No. 17502-048, Thermo Fisher Scientific, USA) under 5% CO2/95% air. After the cells reached confluence, they were shaken for 16 h at 200 rpm to remove the microglial cells and oligodendrocytes. After that, astrocytes were removed and cryopreserved at − 80 °C as a stock. Before the assays, cryopreserved astrocytes were rapidly thawed for plating in DMEM with 10% heat-inactivated FBS and 1% N-2 medium and transferred to noncoated flasks and then cultured for 3 days. After the cells had grown, astrocytes were replated on poly-L-lysine -coated 96-well plates at a density of 2,000 viable cells per well. Four days after plating, the medium was replaced with medium containing 250 μm dibutyryl-cAMP (Bucladesine sodium; Cat No. 029-16383, FUJIFILM Wako Pure Chemical Corporation, Japan) and cultured for 15 days before the assay. On Day 20, the medium containing DMSO or Compound A was added to the astrocyte-seeded plates. After the cells were incubated for 24 h, the medium was collected as the post-treatment samples. The culture medium containing DMSO or Compound A was used for the pre-treatment samples.

### Quantification of pyruvate, lactate and glucose in the culture medium

The levels of pyruvate, glucose and lactate in the pre- and post-treatment samples were measured by biochemical methods. The pyruvate and lactate were measured using Determiner PA and Determiner LA kits (Minaris Medical, Japan), respectively. Glucose was measured using a Glucose C II-Test Wako kit. The changes of these metabolites in 24 h were calculated as the values of post-treatment samples minus that of the pre-treatment samples.

### Brain PDH activity and lactate levels in CSF of rats

The day before administration, sixteen rats were assigned to four groups so that no bias occurred in the body weight between the groups. The vehicle or Compound A was orally administered to each of the rats. 4 h after administration, blood samples were collected from the tail vein while awake and CSF was collected under anesthesia with 2.5% isoflurane. After waking up from anesthesia, whole brain was collected immediately after decapitation, followed by fast freezing with a freezing-clamp.

Brain PDH activity was determined by the AAT-coupled method following previously reported methods [10]. Briefly, the brains were collected in 4 vol of the homogenization buffer and immediately homogenized with the homogenizer. The homogenate was centrifuged at 10,000 g for 20 min and 10 µL of the supernatants were suspended in 150 µL of the sample buffer to obtain active PDH samples. Twenty microliters of the supernatants were suspended with 20 µL of the activation buffer containing 10 mM of dichloroacetate (DCA, Cat No. 347795, Sigma-Aldrich, USA) and incubated at 37°C for 10 min to completely activate the PDH. Then 20 µL of the incubated samples were diluted with 60 µL of the sample buffer to obtain total PDH samples. To eliminate endogenous acetyl-CoA in the samples, 50 µL of each sample was suspended with 130 µL of the reaction buffer and incubated at 37°C for 15 min. After incubation, 20 µL of 50 mM sodium pyruvate solution was added, and conversion of 4-aminoazobenzene-4’-sulfonic acid sodium salt to acetyl-4-aminoazobenzene-4’-sulfonic acid sodium salt was monitored at 460 nm after incubating for 30 min at 30 °C. Brain PDH activity (% active) was expressed as the ratio between the activity of the active PDH samples and total PDH samples, which was then normalized by protein concentration determined with a BCA protein assay kit.

### 2-DG uptake assay

Vehicle or Compound A was orally administered approximately 16 h before 2-Deoxy-d-glucose (2-DG) administration. Animals were decapitated 2 min after the intravenous injection of 2-DG at a dose of 3 mg/kg. Then the brain was removed, and the right cerebral cortex was dissected. The cortices were homogenized and subsequently heated at 95 °C for 15 min. Following centrifugation (17,360 x g for 15 min at 4 °C) to remove the cell debris, the resulting supernatants were used to measure 2-DG levels. The measurement of 2-DG uptake in the tissue was performed using the 2-deoxyglucose uptake measurement kit (Cosmo Bio, Tokyo, Japan) according to the manufacturer’s instructions, enabling the 2-DG uptake into the cells to be quantified as the amount of 2-deoxyglucose 6-phosphate (2-DG6P) without the interference of the remaining extracellular 2-DG based on the enzymatic photometric method [11].

#### Efficacy study using 5xFAD AD model mice

The effect of Compound A on cognitive function was evaluated using Y-maze, Novel object recognition test (NORT) and Morris water maze (MWM) with 5xFAD mice. We used twenty-three Non-Tg mice and forty-five 5xFAD mice for behavioral assessment, which were assigned into three groups: Non-Tg-vehicle (*n* = 23), 5xFAD-vehicle (*n* = 23) and 5xFAD-Compound A (*n* = 22), including both males and females with approximately equivalent numbers. They were divided into three cohorts and were subject to behavioral testing in a staggered manner. They were treated with vehicle or Compound A mixed into chow (PicoLab Rodent Diet 5053, Inotiv, Indiana, U.S.A.) (Compound A mixed chow contained Compound A at the dose of 30 mg/kg/day) for three months beginning from seven-months of age, when amyloid deposition was well-established. In all behavioral assessments, mice exhibiting abnormal behaviors that precluded testing such as rotations or sickness-like states such as freezing were excluded. After all behavioral testing was completed, animals were anesthetized with isoflurane and transcardially perfused with phosphate-buffered saline (PBS) to perform biochemical analysis using one hemisphere and the other hemisphere was immersion fixed for immunohistochemistry.

The effect of Compound A on neurodegeneration and Aβ pathology was evaluated using an additional cohort. Four Non-Tg mice and seven 5xFAD mice were divided into the same three groups as above. The mice were treated with vehicle or Compound A with food for one month. After the treatment, animals were processed with the same procedure as above. The detailed procedure is described below.

### Behavioral assessment

#### Y-maze

One month after starting treatment with Compound A, the mice were tested with the Y-maze. 21 Non-Tg-vehicle, 19 5xFAD-vehicle and 22 5xFAD-Compound A mice completed testing and were analyzed after excluding mice disqualified due to climbing the walls of the apparatus rather than exploring the maze arms or exhibiting sickness-like immobility. Briefly, they were placed in one of the three arms of the apparatus (7.5 × 38 × 12.5 cm) located in a dim room and were allowed to freely explore three arms for 8 min. All sessions were video recorded. Alternation behavior was defined as consecutive entries into each of all three arms without repeated entries, as on overlapping triplet sets. The analysis was performed in a blinded manner by an independent co-worker based on the recorded videos. Alternation rates were calculated as the ratio of alternation behavior to the (total arm entries – 2) × 100.

### NORT

One month after starting treatment with Compound A, the mice were tested with the NORT. The numbers of mice in each group successfully completing NORT and analyzed was 15 Non-Tg vehicle, 12 5xFAD-vehicle and 14 5xFAD-Compound A. The lower n’s completing reflects failure of recording of one of the three cohorts. The NORT was undertaken using a 40 × 40 × 40 cm open field arena, constructed from white plastic material. A LEGO block and a 25 cm^2^ cell culture flask filled with water colored by black paint were used as the objects. Objects were placed at an equal distance from the arena walls. The placement of the objects was counterbalanced by side and the order of object choice in different sessions was randomly assigned across animals to avoid bias. The arena and all the objects used were cleaned with 10% alcohol between sessions. The NORT consists of 3 days. On day 1, the mice were acclimated to the experimental apparatus in the absence of objects for 10 min. On day 2, they were again allowed to explore the apparatus where the same objects were placed for 10 min. On day 3, the mice were placed in the arena with the same object as the previous day and allowed to explore for 10 min (acquisition trial) and returned to the home cage. 1 h after the acquisition trial, one of the objects were replaced with the novel object, and then the mice were placed and allowed to explore for 10 min (retention trial). Behavior was recorded by a digital camera, and the exploration behavior of the animals was manually analyzed based on the recorded video in a blinded manner. The novelty discrimination index (NDI) was defined as the novel object exploration time divided by the total exploration time in the retention trial.

### Morris water maze

Three months after starting treatment with Compound A, the mice were tested with the MWM. 23 Non-Tg vehicle, 15 5xFAD-vehicle mice and 17 5xFAD-Compound A mice completed the MWM. Excluded mice were unable to complete the test due to severe motor deficits. In MWM test, a circular pool with a diameter of 120 cm, a height of 45 cm and a water depth of 25 cm was used, and a colorless transparent platform with a diameter of 12 cm was placed in position about 1 cm below the water surface. All animals were allowed to swim freely in the pool for 60 s to memorize the location of the platform. A 60 s/trial was performed three times/day for five days in the hidden platform test, and a probe test was performed about 4 h after the last trial on the fifth day. In the probe trial, the pool was divided into four quadrants without setting the platform, and the target area was defined as the quadrant where the platform was placed during the hidden platform test, the percentages of time spent in the target area in 60 s were calculated.

### Immunohistochemistry

Mice were anesthetized and transcardially perfused with phosphate-buffered saline (PBS). The right hemisphere was fixed in 4% paraformaldehyde and cryoprotected by immersion in 30% sucrose for 3 days at 4˚C for immunohistochemistry. The serial coronal sections (10 μm) obtained with a cryostat were washed three times in PBS, blocked with 5% bovine serum albumin (BSA) and 0.1% Triton X-100 in PBS for 1 h at room temperature. The primary antibodies include: anti-NeuN (1:100, Cat No. ab177487, Abcam) and anti-amyloid beta (6E10) (1:500, Cat No. 803001, Biolegend) at 4˚C overnight. After washing, slices were incubated with secondary antibodies: anti-rabbit 488 (1:3,000, Cat No. A-11004, invitrogen) and anti-mouse 594 (1:3,000, Cat No. A-11005, invitrogen) at room temperature for 2 h. Images were acquired using an FV-3000 Laser-scanning confocal microscope (Olympus).

Quantitative analysis of NeuN + cells was performed on three sections per animal and analyzed in a blinded fashion by ImageJ software (National Institutes of Health (NIH), Bethesda, MD, USA). Analysis was done in the following steps: (1) images are converted to 8 bit; (2) following converting, selected automatic thresholding method for area of NeuN + cells and background signals were removed; (3) the thresholded images for the designated brain area were quantified using Olympus software and the Analyze particles tool for the “count” value of the NeuN + cells; (4) “count” value of the NeuN + cells was then divided by the designated area. Finally, (5), to normalize relative to the control, the following equations are applied: % of control = (Non-Tg or 5xFAD vehicle or treatment/ average of Non-Tg) x 100. The steps for the quantitative analysis of Aβ were the same up to (2). In (3), the thresholded images for the designated brain area were quantified using the Analyze particles tool for the “%Area” value of the Aβ; (4) to normalize relative to the control, the following equations were applied to the “%Area” values of the two groups to be compared: % of 5xFAD = (%Area 5xFAD-vehicle or 5xFAD-Compound A/ %Area average of 5xFAD) × 100.

### Metabolomic analysis using liquid chromatography-mass spectrometry (LC-MS)

The frozen brain stored at -80 °C was extracted by adding methanol on ice (added 100 µL/10 mg sample) followed by vortexing for 1 min. The homogenates were centrifuged for 5 min (10,000 rpm, 4 °C), and the supernatant was collected. A mixture of 45 µL of supernatant and 5 µL of internal standard solution (Metabolite Yeast Extract (Cat No. ISO1, Cambridge Isotope Laboratories), L-(+)-Lactic Acid-13C3 Sodium Salt (Cat No. L113507, Toronto Research Chemicals Inc.) and Acetylcholine chloride (Cat No. A6625-25G, Sigma)) was used along with brain methanol extracts for metabolomic analysis.

LC-MS analysis was performed using a Nexera UHPLC/HPLC system and a triple quadrupole mass spectrometer LCMS-8060 (Shimadzu, Kyoto, Japan). The LC system was equipped with a ZIC-pHILIC column (2.1 × 150 mm, 3 μm, Sequant, Darmstadt, Germany). Chromatographic separation was based on the previous report [12]. For mobile phases A and B, 20 mmol/L ammonium bicarbonate in water and acetonitrile were used, respectively. The flow rate was 0.25 mL/min. The sample cooler and column oven temperatures were set at 4 °C and 45 °C, respectively. The gradient of mobile phase B concentration was programmed as 90% (0 min) − 90% (5 min) − 30% (20 min) − 30% (23 min) − 90% (24 min) − 90% (35 min). Sample injection volume was 5 µL. The parameters for the mass spectrometer were set as follows: nitrogen was used for nebulizer gas, drying gas, and heating gas. The flow rate of the nebulizer-, drying-, and heating gas were set at 3 L/min, 10 L/min, and 10 L/min, respectively. Argon was used for collision-induced dissociation (CID). The heat block, desolvation line, and interface temperatures were set at 400 °C, 250 °C, and 300 °C, respectively. Mass spectrometric data was acquired by multiple reaction monitoring (MRM). MRM transitions were based on the software method package ver.1 for the simultaneous analysis of primary metabolites (Shimadzu, Kyoto, Japan).

Traverse MS ver. 1.2.9 (Reifycs, Tokyo, Japan) was used for the data processing, including peak detection and integration, and calculation of peak areas. The peak area of each metabolite was divided by that of corresponding internal standard to calculate relative levels of metabolites. Heatmap visualization of the z-scored levels of metabolites in cerebral cortex was performed using the ggplot2 package using the R software.

### Statistical analysis

The results were expressed as means ± standard deviations. Data generated from the above studies were statistically analyzed with GraphPad Prism (version 6.07). Comparisons between the two data sets were performed by unpaired t-test or one-way ANOVA, followed by Dunnett-test. Correlation analysis was performed by linear-regression analysis followed by Pearson test. A P value of < 0.05 was considered statistically significant.

## Results

### Inhibitory activity of compound A

**Fig. 1:**
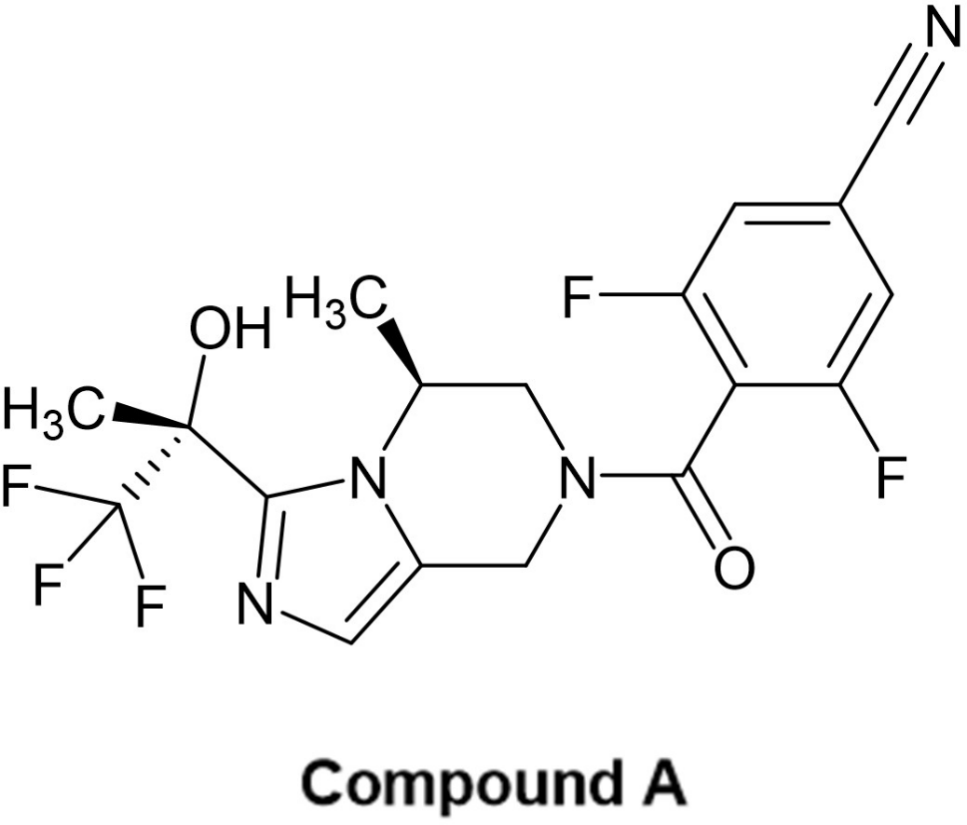
Chemical structure of compound A

We used a novel, orally available small-molecule PDHK inhibitor Compound A, which we identified at the Chemical Research Laboratories at Japan Tobacco Central Pharmaceutical Research Institute (Fig. 1). The inhibitory activity of Compound A was evaluated by measuring residual PDH activity as described in the Materials and Methods section. It revealed that Compound A potently inhibited hPDHK1, 2 and 3 with IC_50_ of 8.17 ± 0.56 nM, 5.33 ± 0.12 nM, and 50.5 ± 3.23 nM, respectively, while it did not inhibit hPDHK4 up to the concentration of 10 µM. The specificity was confirmed by demonstrating no inhibitory activity on other kinases and other major enzymes, receptors and transporters that are expressed in the central nervous system even at the highest concentration tested (10 µM, data not shown).

### Effect of compound A on glucose metabolism in primary CNS cells

To understand how Compound A affects glucose metabolism in brain cells, we first investigated the effect of Compound A on glucose metabolism with primary neurons and astrocytes which are major interacting compartments in brain glucose metabolism. The changes in levels of pyruvate, lactate and glucose in the media of the cell cultures after treatment with Compound A for 24 h were evaluated.

Both in the primary neurons and astrocytes, the level of pyruvate was reduced in the vehicle-treated cells after 24 h (Fig. 2A, D), suggesting that PDH activity was increased by Compound A in both cell types. A significant difference between the treatment of vehicle and Compound A was observed at concentrations above 0.1 µM of Compound A in neurons (Fig. 2A) and 0.01 µM of Compound A in astrocytes (Fig. 2D). In the primary neurons, the level of glucose remained unchanged by cells with vehicle or Compound A treatment over 24 h (Fig. 2B). The lactate level in the vehicle treated cells was also not altered over 24 h, however, it was significantly reduced by Compound A treatment (*p* < 0.05 at 1 µM compared to vehicle) (Fig. 2C). In contrast to neurons, in the primary astrocytes, the level of glucose was reduced in the vehicle-treated cells after 24 h (Fig. 2E). A significant difference between the treatment of vehicle and Compound A was observed at concentrations above 0.01 µM of Compound A. In the same cultures, the level of lactate was increased in the vehicle-treated cells during 24 h (Fig. 2F). From these experiments, we conclude that in vitro Compound A has the effect of enhancing glucose consumption and lactate production in astrocytes, as well as enhancing lactate consumption by neurons.

### In vivo pharmacological profile of compound A

Next, to confirm whether Compound A exerts the same effects in the adult brain, we evaluated the effect of Compound A on the PDH activity in adult rat brain in vivo. Pharmacokinetic measurements revealed that Compound A has a good oral bioavailability with close to 100% absorption as well as the suitable pharmacokinetic profile for in vivo use (C_max_ brain = 0.50 µM, T_max_ = 4.0 h, T_1/2_ = 9.1 h, after oral administration of Compound A at a dose of 3 mg/kg to SD rats, data not shown).

**Fig. 3:**
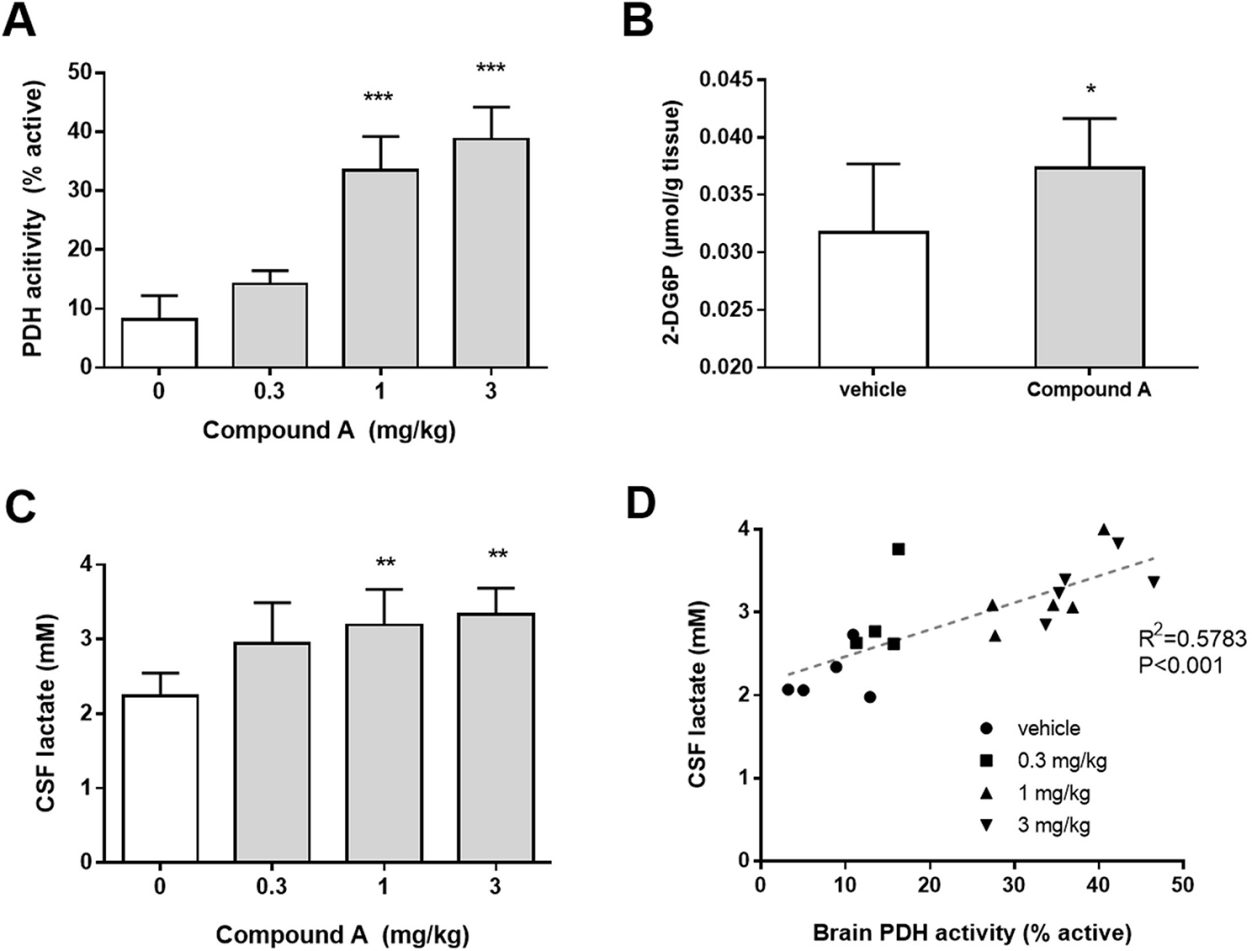
Compound A increased aerobic glycolysis by increasing PDH activity in the brain. PDH activity **(A)**, 2-DG uptake **(B)** in the brain and the lactate levels in the CSF **(C)** in rats after single oral administration of Compound (A) **(D)** The correlation analysis between the brain PDH activity and the CSF lactate levels. Data were analyzed by one-way ANOVA followed by Dunnett-test for A and C, by Student’s t-test for (B) Correlation analysis was performed by linear-regression analysis followed by Pearson test for D. *p < 0.05, **p < 0.01, ***p < 0.001 vs. vehicle. *n* = 4–5 for A and C, *n* = 8–9 for B, respectively. Data are shown as the mean ± S.D

The rats were orally administered Compound A and were sacrificed after 4 h, corresponding to the Tmax. The brain PDH activity was increased by Compound A. A significant increase compared to vehicle was observed at doses above 1 mg/kg (Fig. 3A). In addition, we examined the effects of Compound A on glucose uptake in the brain as well as the lactate levels in CSF, as a measure of glucose metabolism (glycolysis) to see if Compound A recapitulates the findings of our in vitro experiments in terms of glucose metabolism. Because this system enabled us to evaluate the glucose uptake more accurately than other systems such as [^3^H]2-DG, we evaluated the effect of Compound A on glucose uptake in the cerebral cortex by measuring 2-DG6P, a metabolite of 2-DG that is produced after 2-DG is taken up by cells. Compound A, at a dose of 3 mg/kg, significantly increased 2-DG6P accumulation in the cortex as shown in Fig. 3B. We checked whether Compound A affected the plasma glucose level because the uptake of 2-DG may be affected by plasma glucose levels. We found that it was not affected by Compound A (Figure S1), demonstrating the improved brain glucose uptake induced by Compound A. Moreover, the level of lactate in CSF was increased (Fig. 3C). Interestingly, we found that the PDH activity was positively correlated with the lactate level in CSF (Fig. 3D). These results demonstrated that Compound A increased glycolysis via PDH activation in vivo.

### Evaluation in AD model mice: behavioral tests

**Fig. 4:**
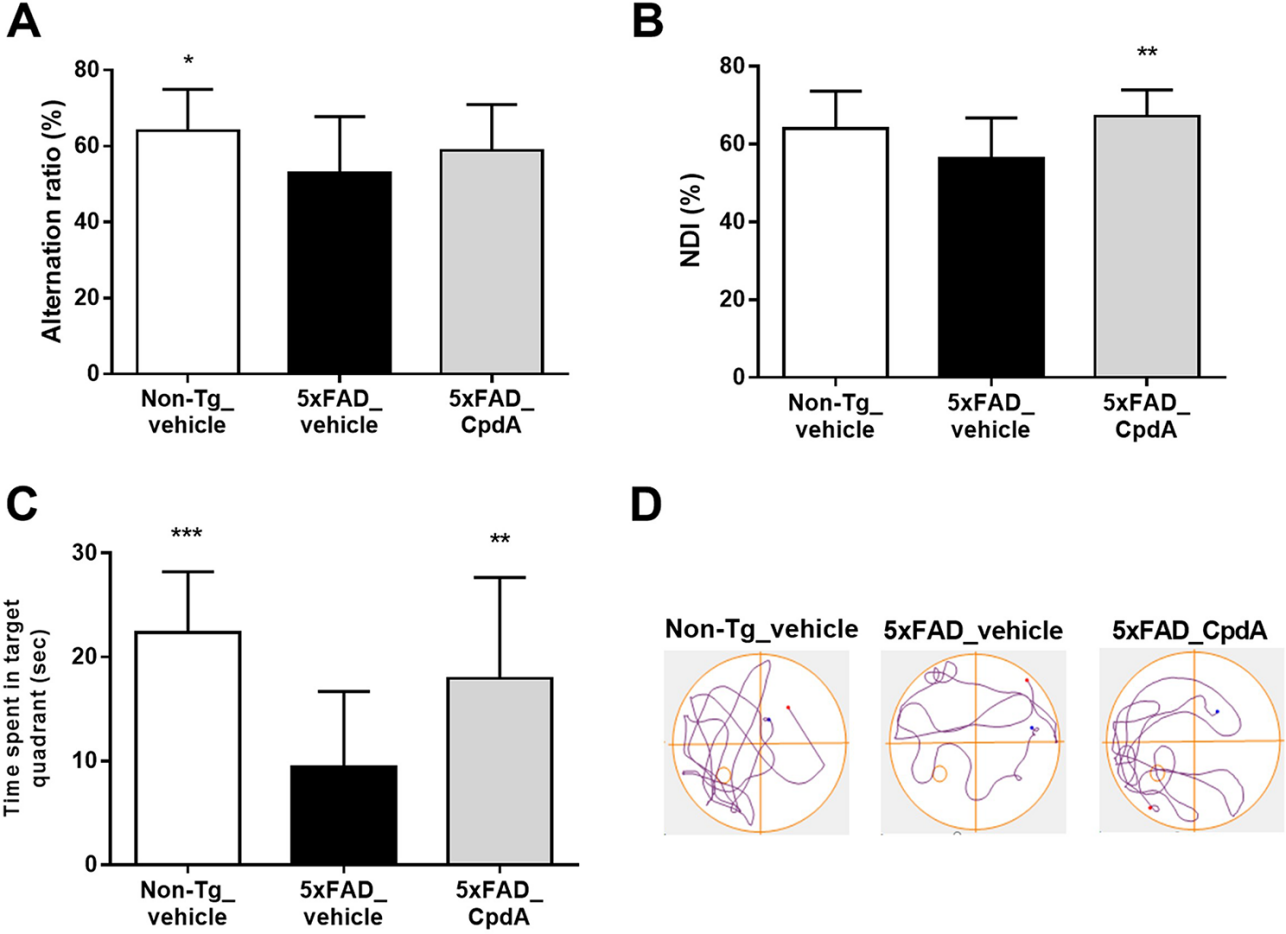
Compound A ameliorated cognitive dysfunction in 5xFAD mice. The cognitive function was evaluated by measuring alternation behavior in Y-maze test **(A)**, NDI in NORT **(B)** and the time spent in the target area in MWM **(C)**. The representative trace of each group during the probe test in MWM are shown in **(D)**. Data were analyzed by one-way ANOVA followed by Dunnett-test. *p < 0.05, **p < 0.01, ***p < 0.001 vs. 5xFAD-vehicle. *n* = 17–23 for A and C, *n* = 12–15 for B, respectively. Data are shown as the mean ± S.D

First, we performed Y-maze to investigate the effect of Compound A on spatial working memory after 1-month treatment. The vehicle-treated 5xFAD mice showed a deficit in spatial working memory (17.4% reduction in alternation behavior, *p* < 0.05, Fig. 4A) compared to the vehicle-treated Non-Tg mice. Compound A showed a trend for improvement (*p* = 0.176). There was no difference in the number of total arm entry among all the experimental groups, confirming that changes in alternation behavior were not due to impaired exploratory or spontaneous locomotor activities (Figure S2A).

Next, NORT was performed to investigate the effect of Compound A on the short-term memory. The NDI of the vehicle-treated 5xFAD mice was reduced compared to that of vehicle-treated Non-Tg mice (11.8% reduction, *p* = 0.062) although it was not significant due to the wide inter-individual variation (Fig. 4B). The NDI of the Compound A-treated 5xFAD mice was significantly higher than that of vehicle-treated 5xFAD mice (*p* < 0.01). To confirm the decrease in NDI of the vehicle-treated 5xFAD mice was not due to decreased exploration behavior, the total exploration time was calculated. No difference was observed among all experimental groups (Figure S2B).

Lastly, the effect of Compound A on the spatial memory was evaluated by the MWM test including a probe test in 5xFAD mice. The time spent in the target area, an index of cognitive function, was significantly lower in the 5xFAD mice compared to that in the Non-Tg mice, indicating that 5xFAD mice displayed cognitive dysfunction (Fig. 4C). The time spent in the target area was significantly higher in the Compound A-treated 5xFAD mice compared to the vehicle-treated 5xFAD mice while the total distance traveled during the probe test was not different between the treatments (Figure S2C). Figure 4D shows the representative traces of each group during the probe test. These results from behavioral assessment indicates that Compound A ameliorated cognitive dysfunction in 5xFAD mice.

### Immunohistochemistry: neuron loss

**Fig. 5:**
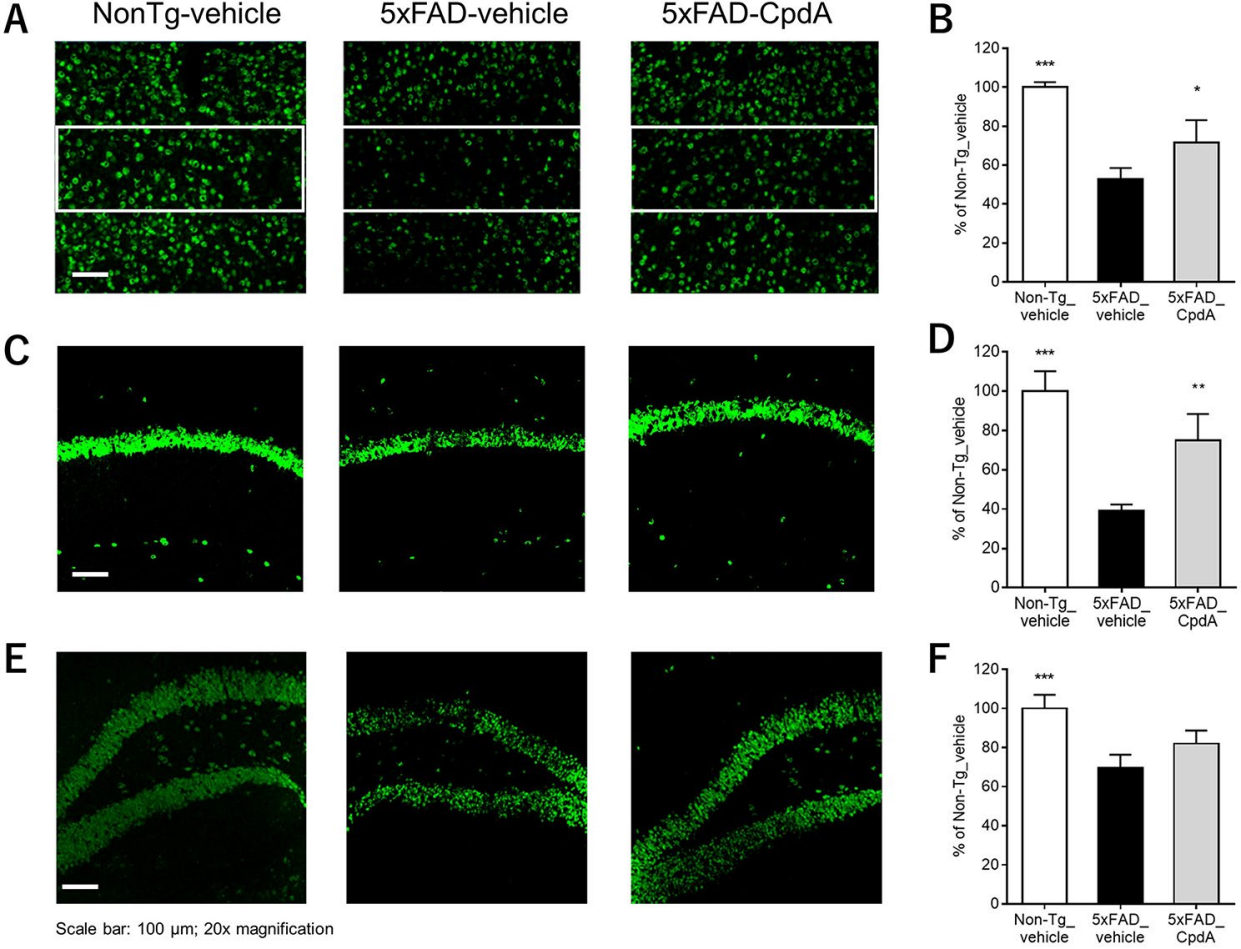
Effect of Compound A on neuron loss in 5xFAD mice. Immunofluorescence for NeuN in the cortex layer 5 (surrounded by a white box) **(A)**, hippocampal CA1 **(C)** and DG **(E)** of 5xFAD mice. Quantification of NeuN + counts in the cortex layer 5 **(B)**, hippocampal CA1 **(D)** and DG **(F)**. Data were analyzed by one-way ANOVA followed by Dunnett-test. *p < 0.05, **p < 0.01, ***p < 0.001 vs. 5xFAD-vehicle. *n* = 3–4. Data are shown as the mean ± S.D

The effect of Compound A on neuron loss in the hippocampus CA1, DG and layer 5 of the cerebral cortex was evaluated in 5xFAD mice by immunohistochemistry. The number of NeuN-positive cells, an index of living neurons, was significantly lower in the 5xFAD mice compared to the Non-Tg mice, indicating 5xFAD mice exhibited neuron loss at approximately 11 months of age. This is consistent with published data [13]. In contrast, the number of NeuN-positive cells was significantly higher in the Compound A-treated 5xFAD mice compared to that in the vehicle-treated 5xFAD mice (Fig. 5). We confirmed this neuroprotective effect persists after three months of treatment using a different in-house PDHK inhibitor distinct from Compound A (data not shown), suggesting the effect above is not a temporary delay or off-target effect. These results indicate that Compound A protected against neuron loss observed in 5xFAD mice.

#### Immunohistochemistry: Aβ deposits

**Fig. 6:**
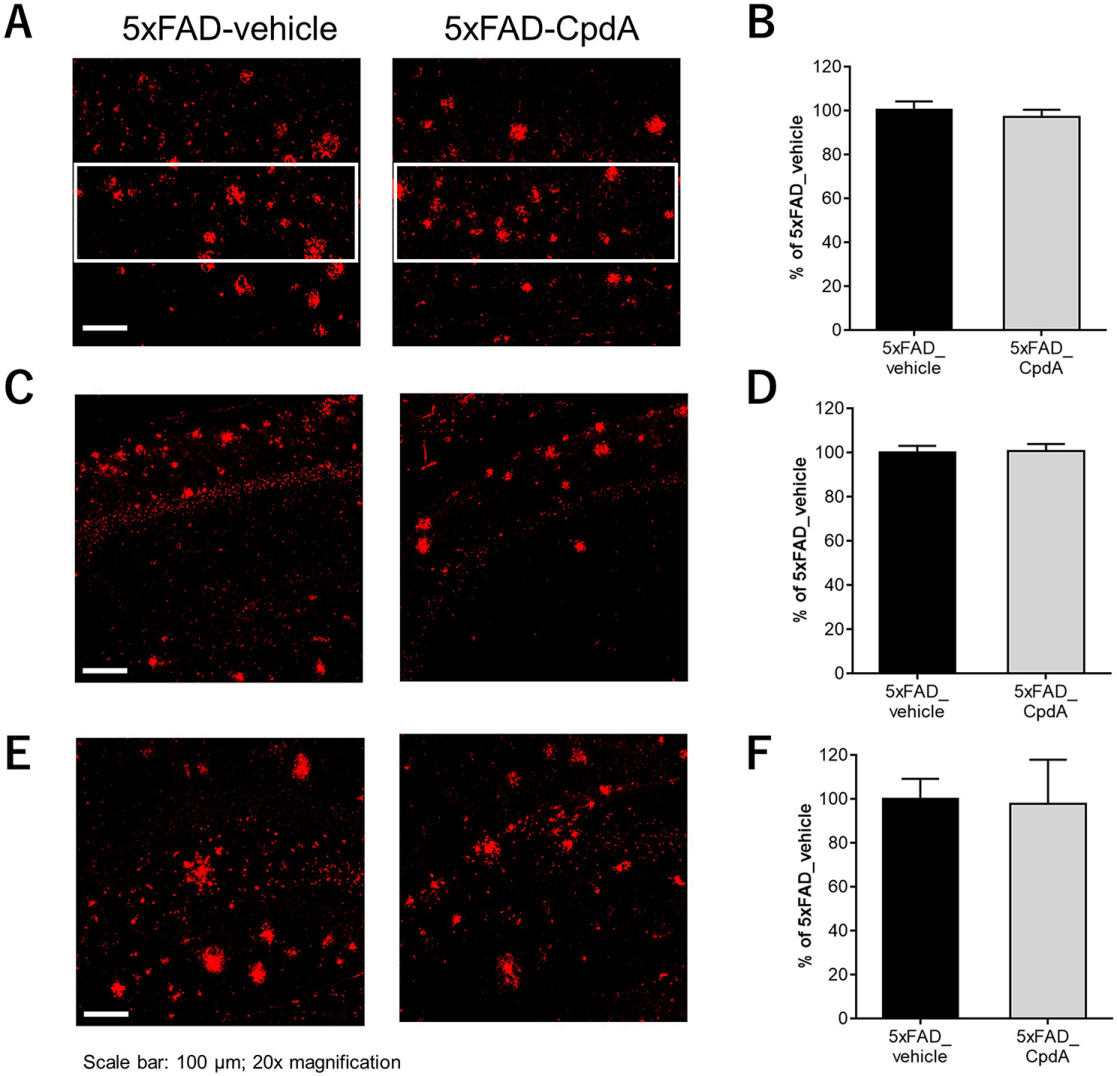
Lack of effect of Compound A on Aβ deposits in 5xFAD mice. Immunofluorescence for Aβ in the cortex layer 5 (surrounded by a white box) **(A)**, hippocampal CA1 **(C)** and DG **(E)** of 5xFAD mice. Quantification of Aβ-positive area in the cortex layer 5 **(B)**, hippocampal CA1 **(D)** and DG **(F)**. *n* = 3–4. Data are shown as the mean ± S.D.

To understand whether Compound A prevents cognitive deficits and neuron loss via limiting Aβ accumulation, we evaluated the effect of Compound A on Aβ deposits in the hippocampal CA1, DG and in the layer 5 of cerebral cortex in 5xFAD mice by immunohistochemistry. As shown in Fig. 6, extensive Aβ deposits were detected by 6E10 antibody in all areas observed in the brains of 5xFAD mice. However, Compound A had no effect on Aβ deposits in all examined regions in 5xFAD mice. This was also replicated with different PDHK inhibitor and 3 months treatment in an independent cohort (not shown).

### Effects of compound A on glucose metabolism in the brain of 5xFAD mice

**Fig. 7:**
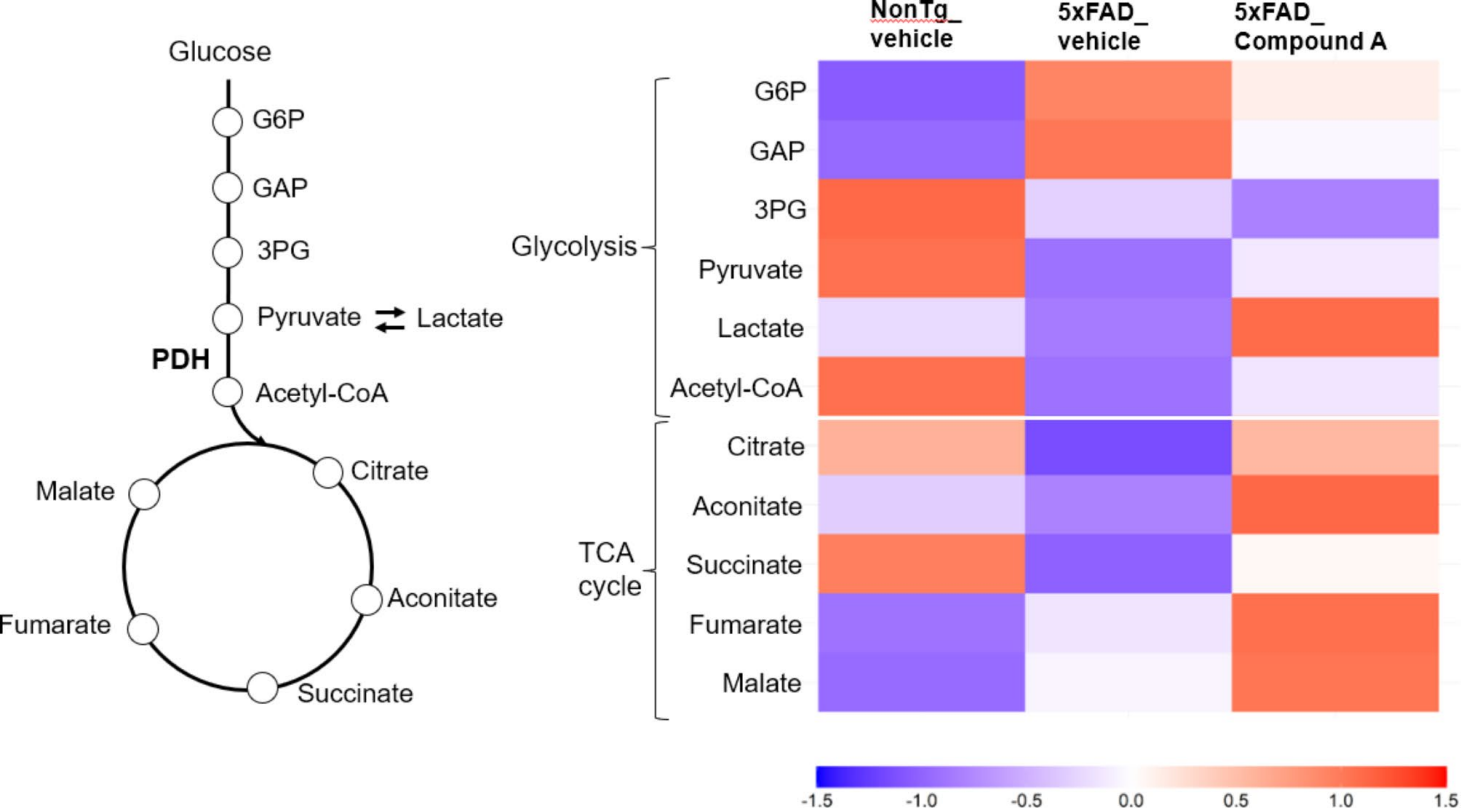
Heatmap of glycolysis- and TCA cycle-related metabolites in the cerebral cortex from 5xFAD mice. It is displayed as the z-score of averaged levels. The red, white and blue color indicate positive, zero and negative z-score, respectively. *n* = 9–10. Data were analyzed by one-way ANOVA followed by Dunnett-test

To investigate whether Compound A may have exerted the observed protective effects in 5xFAD through improving glucose metabolism in the brain, we measured glucose metabolism-related metabolites in the cerebral cortex of Non-Tg mice and 5xFAD mice treated either with vehicle or Compound A using metabolomics. The results showed that, compared to Non-Tg mice, 5xFAD mice exhibited impaired glucose metabolism as indexed by a dramatic increase in the initial metabolites of glycolysis including glucose-6-phosphate (G6P; *p* < 0.01) and Glyceraldehyde-3-phosphate (GAP; *p* < 0.001) but a decrease in the downstream metabolite pyruvate (*p* < 0.01) as well as TCA cycle-related metabolites, suggesting reduced PDH activity (Fig. 7). In the Compound A-treated 5xFAD mice, these altered levels of metabolites tended to be normalized; the initial or upper metabolites showed a trend for decrease while the lower metabolites increased (e.g. pyruvate; *p* < 0.05). In addition, the metabolites downstream of PDH including acetyl-CoA were overall increased although the effects were not all significant due to a wide inter-individual variability. These results suggest that the impaired glucose metabolism observed in 5xFAD mice was improved by treatment with Compound A.

## Discussion

In the present study, we revealed that Compound A is an orally active, potent CNS-acting PDHK inhibitor. In addition, to the best of our knowledge, we report for the first time that inhibition of PDHK leads to an increase of glucose uptake and increased CSF lactate levels. Furthermore, the findings from the efficacy study with 5xFAD model indicate that Compound A improved cognitive impairment and limited neuron loss in 5xFAD mice without reducing Aβ deposits. Our results showed that selectively treating deficits in glucose metabolism led to prevention of neuron loss and cognitive decline even after amyloid deposition was well-established. These results argue that the metabolic deficits that parallel neurodegeneration in AD may not simply be a consequence of reduced glucose demand due to neuron and synapse loss but are actually a treatable, causal and important contributing factor.

### Compound A as a potent PDHK inhibitor

Compound A was found to inhibit human PDHK1-3 but not PDHK4. We confirmed that Compound A shows a similar inhibitory profile on rat PDHKs (data not shown). It is reasonable that no species differences were observed given the very high protein homology between mouse, rat and human PDHKs (~ 98%, based on the NCBI Homologene database). Compound A was also found to have a good PK brain profile capable of markedly increasing PDH activity in the rat brain, demonstrating that Compound A is a potent CNS-active PDHK inhibitor.

### Pharmacological profile of compound A

From our experiments using primary CNS cell cultures, Compound A increased the consumption of pyruvate both in neurons and astrocytes, indicating that PDH activity was increased by Compound A treatment. Compound A also increased the neuronal consumption of lactate by activating PDH. In contrast, and surprisingly, Compound A enhanced the lactate production as well as glucose uptake in astrocytes. These results indicate that Compound A activated aerobic glycolysis (AG). AG is a non-oxidative metabolism of glucose despite the presence of abundant oxygen that accounts for 10–12% of glucose used by the adult human brain [14]. Although the mechanism by which Compound A activated AG remains unknown, a recent work by Wang et al. (2022) provides us with a clue for speculating on the mechanism connecting PDH activation by Compound A and enhancement of AG. Astrocytes have constitutively high pPDH but have a lower capacity for recycling NADH to NAD^+^ in mitochondria which activates glycolysis to maintain the recycling of NAD^+^ when it is saturated. Since activation of PDH by Compound A also enhances consumption of NAD^+^, astrocytes may enhance glycolysis (AG) in response to Compound A via this Warburg effect [15]. In contrast, in normal neurons which are more dependent on oxidative phosphorylation, PDH phosphorylation at the PHDK site in PDH (the ser293 target site) is suppressed by high neuronal activity in vitro and in vivo which in turn upregulates high neuronal PDH activity and pyruvate utilization by PDHC which limits downstream conversion to lactate and therefore lower lactate production [16].

Our collective observations of Compound A effects on neurons and astrocytes support the Astrocyte-Neuron Lactate Shuttle (ANLS) hypothesis. The ANLS hypothesis originally proposed by Pellerin and Magistretti is a model postulating that neurons are predominately oxidative and astrocytes predominately glycolytic cells which implies intercellular lactate transfer from astrocytes to neurons with further oxidation of lactate in neurons through the TCA cycle [17], see review [18]. Although this hypothesis is controversial because there are many papers claiming the opposite findings including direct utilization of glucose by neuronal cells [19, 20], our in vitro and in vivo findings lead us to the working hypothesis that Compound A activates the ANLS. Supporting this hypothesis, profiling of Compound A in vivo has revealed that Compound A increased glucose (2-DG) uptake in rat brain and increased lactate levels in CSF while also increasing PDH activity. These results indicate that Compound A activates ANLS *in vivo.*

The finding of an enhancement of glucose uptake in the brain and increased lactate levels in the CSF by inhibition of PDHK is a first report in the area of PDHK-related research and in contrast to the previous reports with DCA, a classical PDHK inhibitor, which decreased CSF lactate levels in humans [21], see review [22], and AD model mice [23]. In our own experiments with DCA, we have confirmed the decrease in CSF lactate after a single administration of DCA in SD rats (unpublished data). Further study will be needed to understand this discrepancy which may reflect the difference in their inhibitory profiles. In any event, in increasing extracellular lactate available to neurons, Compound A has a beneficial effect consistent with activating ANLS.

### Efficacy of compound A in 5xFAD mice

We explored the effects of Compound A on cognitive function using 5xFAD model mice. The 5xFAD model is a transgenic mouse model used to study AD-related pathophysiology induced by severe amyloid burden. 5xFAD mice exhibit impaired glucose metabolism as demonstrated in this study and other studies using FDG-PET [24]. This model also shows decreased PDH activity as observed by increased phosphorylated PDH E1α in the brain [25]. With this model, we demonstrated that Compound A improved the impairments in NORT and MWM but only showed a trend to improve Y-maze due to wide inter-individual variability, a recognized problem in the 5xFAD model [26]. As is widely known, these behavioral tests reflect cerebral cortex and/or hippocampal-dependent tasks [27] and are all impaired in 5xFAD mice. Consistent with limiting cognitive decline, Compound A treatment significantly attenuated the neuron loss observed in 5xFAD compared to vehicle treatment. Metabolic deficits sensitize neurons to multiple regulated cell death pathways [28] that have been implicated in AD [29], including necroptosis in 5xFAD and AD [30], recently shown to be downstream from tauopathy in human neurons [31]. Interestingly, Compound A did not affect the Aβ burden. This contrasts with the recent work by Parkin et al. who reported that DCA enhanced sAPPα generation and inhibited the generation of Aβ-peptides in SH-SY5Y neuroblastoma cells [32]. One possibility for this discrepancy is the difference of inhibitory profiles between these two compounds. Alternatively, reducing Aβ production at 7 months when 5xFAD have robust hippocampal and cortical layer 5 Aβ may have diminished impact on well-established Aβ deposits. Compound A would not be predicted to improve metabolic and related functional deficits in microglial amyloid clearance, because microglia primarily express PDHK4, the isoform not targeted by Compound A.

Our findings that Compound A ameliorated the cognitive impairments and prevented neuron loss independent of Aβ plaque pathology in 5xFAD mice indicate that the amelioration of glucose metabolism may be beneficial beyond Aβ-reducing therapies and a useful primary or adjunct intervention, especially in patients who already have extensive Aβ pathology.

In the mechanistic aspect, we found that Compound A normalized the impaired glucose metabolism of 5xFAD. In the vehicle-treated 5xFAD mice, the upper metabolites of glycolysis such as G6P was significantly increased while the lower metabolites including TCA cycle were decreased compared to Non-Tg mice (Fig. 7). This indicates the flow of glycolysis may be impaired in the diseased brain. There are several papers supporting the relevance of our findings in these AD model mice to postmortem brains from AD patients which exhibit higher brain tissue glucose concentration, reduced glycolytic flux, and lower GLUT3 associated with the severity of AD pathology [33] as well as decreased GAPDH and glycolytic activity in the AD brain [34]. Our observations with Compound A’s improvement of abnormal glucose metabolism of 5xFAD mice combined with compelling evidence for PDH activity deficits in AD indicate that this compound may well normalize the impaired glucose metabolism in AD brains as well. One caveat is that the 5xFAD model lacks tauopathy comparable to AD while isolated tauopathy in mice also impairs metabolism in the absence of Aβ.

### Potential of PDHK inhibitor as a treatment of AD

In the brains of AD, the PDH activity has been reported to be reduced as cited above. Similar PDH deficits have been reported in another AD mouse model as decreased PDH activity was reported in 3xTg [35] and PDH with increased inhibitor phosphorylation was found in 5xFAD [25]. Although the mechanisms responsible for reduced PDH activity in AD and AD models remain unclear, one proposed pathway is that the expression of PDHKs may be increased by HIF-1 upregulation in the brains of AD patients [36, 37]. Under various hypoxic conditions including aging [38], AD, and more typically cerebral ischemic injury [39], HIF-1 is induced and plays a role in attenuating the consumption of oxygen by inducing PDHKs to reduce oxidative phosphorylation [40, 41]. Finally, stress and depression, two risk factors for AD [42, 43], increase glucocorticoid-mediated induction of CNS PKHK2, phosphorylation of PDH and metabolic deficits [44]. Considering this accumulating evidence, it is likely that the PDHK activity and/or the expression of PDHK is pathologically enhanced in the brains of AD and vascular dementia patients where it contributes to reduced PDH activity and metabolic deficits.

Substantial evidence has demonstrated that glucose metabolism including AG and mitochondrial oxidative phosphorylation are broadly impaired in AD (see review [45]). In the diseased neurons, pyruvate derived from lactate and/or glucose is insufficiently converted into acetyl-CoA and energy due to impaired mitochondrial function. Thus, decreased PDH activity can lead to both acetyl-CoA and energy shortages. These deficits may contribute to the dysfunction of neurons and/or subsequent neuron loss. Activation of ANLS is expected to protect neurons from death and to improve their functions by increasing the lactate supply from astrocytes, i.e., AG. For example, lactate treatment ameliorates neurological deficits after ischemia in the transient ischemia model [46]. The recent evidence on neuroprotective roles of lactate is summarized by Mason [47]. Consistent with this protective mechanism, our neuroprotective Compound A increased lactate production.

Because impaired glucose metabolism including reduced neuronal uptake, impaired AG and suboptimal TCA occurs early and at preclinical stages in humans developing AD, there have been multiple approaches to target brain energy deficits in preclinical models. These include improving oxidative phosphorylation, treating with insulin or other diabetes drugs that increase insulin sensitivity, as well as small molecules that correct mitochondrial dysfunction, ketone-based interventions, RNA therapeutics and multimodal lifestyle changes [48]. While mouse models, including 5xFAD, recapitulate not all the features of AD, some of the preclinical efforts to treat brain energy deficits have already advanced to produce promising benefits in clinical trials. Notably lifestyle interventions exemplified by the FINGER trial, some diabetes treatments (metformin and incretin receptor agonists) and the ketone-based approaches which are all moving forward in ongoing and planned trials. In particular, modest, but clinically significant increases in cognitive function have been reported in multiple small trials in mild cognitive impairment (MCI) and mild AD patients with ketogenic supplements and diets. These treatments increase ketone body production to deliver acetyl-CoA to neurons, effectively bypassing defective AG [49]. Further, consistent with reports that functional connectivity and associated cognitive decline are critically sensitive to hypometabolism [50, 51], a 6 month long ketogenic supplement improved cognition and underlying functional connectivity and axonal integrity in patients with MCI [52]. While supporting a causal role for metabolic deficits, the significant but still modest effects of ketone supplements and concerns about the long-term impact of high dose saturated fats on lipid profiles and cardiovascular risk suggest the importance of exploring potentially more potent and direct treatment of the underlying defective glucose metabolism.

In conclusion, considering both the pathophysiology of AD described above and the in vitro/ in vivo profiles of Compound A revealed in this study including activating AG/ANLS and enhancing pyruvate utilization, activating PDH through inhibiting PDHK by Compound A is expected to be a promising therapeutic strategy for AD.

**﻿Fig. 2:**
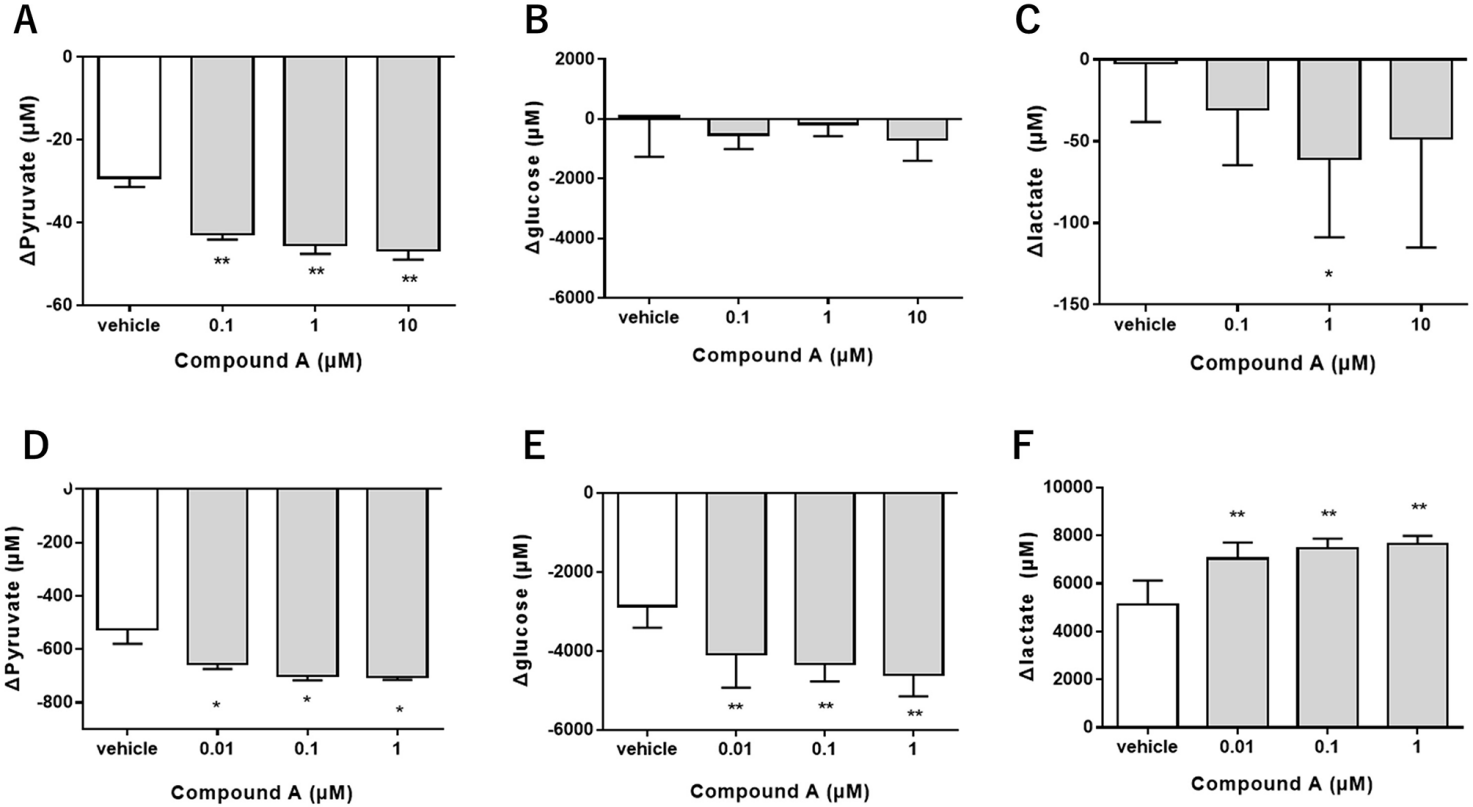
Compound A altered glucose metabolism differentially in primary neurons and astrocytes. The cells were treated with Compound A for 24 h and the changes in the levels of metabolites in the media of cells. **A-C** Changes in pyruvate **(A)**, glucose **(B)** and lactate **(C)** levels in the media of primary neurons. **D-F** Changes in pyruvate **(D)**, glucose **(E)** and lactate **(F)** levels in primary astrocytes. Data were analyzed by one-way ANOVA followed by Dunnett-test. Data are shown as the mean ± S.D. *p < 0.05, **p < 0.01

## Electronic supplementary material

Below is the link to the electronic supplementary material.

Supplementary Material 1

## Data Availability

The datasets generated and analyzed during the current study are not publicly available due to confidentiality policy of the company but are available from the corresponding author on reasonable request.

## Abbreviations

Aβ: Amyloidβ
Acetyl-CoA: Acetyl-Coenzyme A
AG: Aerobic glycolysis
AD: Alzheimer’s Disease
ANLS: Astrocyte-neuron lactate shuttle
ATP: Adenosine tri-phosphate
CSF: Cerebrospinal fluid
DCA: Dichloroacetate
KGDH: α-ketoglutarate dehydrogenase
MCI: Mild cognitive impairment
MDH: Malate dehydrogenase
MWM: Morris water maze
NDI: Novelty discrimination index
NMDA: N-methyl-D aspartate
NORT: Novel object recognition test
PDH: Pyruvate dehydrogenase
PDHK: Pyruvate dehydrogenase kinase
SDH: Succinate dehydrogenase
TCA: Tricarboxylic acid
2-DG: 2-Deoxy-d-glucose
2-DG6P: 2-Deoxy-d-glucose-6-phosphate
