# Supplementary material for: A novel PDHK inhibitor restored cognitive dysfunction and limited neurodegeneration without affecting amyloid pathology in 5xFAD mouse, a model of Alzheimer’s disease
Source: Alzheimers Res Ther. 2024 Sep 5;16:197. doi: 10.1186/s13195-024-01552-2 (PMC11376040; doi:10.1186/s13195-024-01552-2)
Supplement: Supplementary file 1 — Supplementary Material 1 [file 13195_2024_1552_MOESM1_ESM.docx]

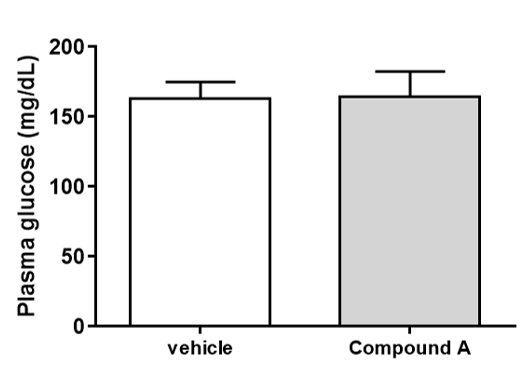
**Figure S1** Effect of Compound A on plasma glucose levels in rats in the 2-DG uptake experiment. Data are shown as the mean ± S.D.


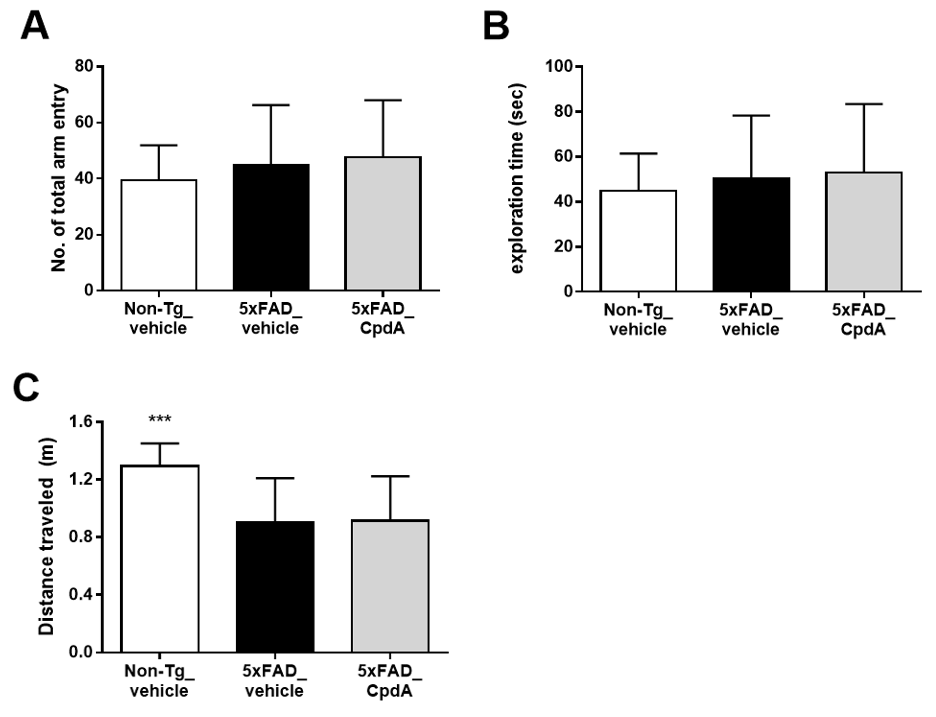


**Figure S2** Effect of Compound A on the total arm entry in Y-maze test (A), exploration time in NORT (B), and the distance traveled during the probe test in MWM (C). Data were analyzed by one-way ANOVA followed by Dunnett-test. ***p<0.001 vs 5xFAD-vehicle. Data are shown as the mean ± S.D.
